# Clinical Effectiveness of Hypofractionated Proton Beam Therapy for Liver Metastasis From Breast Cancer

**DOI:** 10.3389/fonc.2021.783327

**Published:** 2021-11-03

**Authors:** Tae Hyun Kim, Keun Seok Lee, Sung Hoon Sim, Yeon-Joo Kim, Dae Yong Kim, Heejung Chae, Eun-Gyeong Lee, Jai Hong Han, So Youn Jung, Seeyoun Lee, Han Sung Kang, Eun Sook Lee

**Affiliations:** ^1^ Center for Proton Therapy, National Cancer Center, Goyang, South Korea; ^2^ Center for Breast Cancer, National Cancer Center, Goyang, South Korea

**Keywords:** liver metastasis, breast cancer, freedom from local progression rate, overall survival, proton beam therapy, radiotherapy

## Abstract

**Background:**

Few studies of proton beam therapy (PBT) for patients with liver metastasis from breast cancer (LMBC) are available to date. The aim of the present study was to evaluate the clinical effectiveness of PBT for patients with LMBC.

**Material and Methods:**

Seventeen patients with LMBC treated with PBT were included in this study. The median prescribed dose of PBT was 66 GyE (range, 60–80) in 10 fractions, 5 times a week. In patients with LMBC receiving PBT, freedom from local progression (FFLP), progression-free survival (PFS), and overall survival (OS) rates were assessed.

**Results:**

The median follow-up time was 34.2 months (range, 11.5–56.1). The median FFLP time was not yet reached, and the 3-year FFLP rates were 94.1% (95% confidence interval [CI], 82.9–105.3). The median times of PFS and OS were 7.9 months (95% CI, 5.3–10.5) and 39.3 months (95% CI, 33.2–51.9), respectively, and the 3-year PFS and OS rates were 19.6% (95% CI, -1.8–41.0) and 71.7% (95% CI, 46.8–96.6), respectively. Grade 3 or higher adverse events were not observed.

**Conclusion:**

PBT for patients with LMBC showed promising FFLP and OS with safe toxicity profiles. These findings suggest that PBT can be considered a local treatment option in patients with LMBC.

## Introduction

Patients with liver metastases from breast cancer (LMBC), considered a manifestation of incurable systemic disease, have a poor prognosis of 4–8 months’ survival, if untreated, and 18-24 months, even with systemic chemotherapy and/or hormonal treatments (1). The role of local treatments, including hepatic resection, is controversial in patients with LMBC, but hepatic resection is still a potentially curative treatment and could increase survival in selected patients who have metastatic disease confined within the liver or stable extrahepatic disease after systemic treatments (2, 3). However, most patients with LMBC remain ineligible for hepatic resection, which has raised the need for other local treatments, such as radiofrequency ablation (RFA), cryoablation, transarterial chemoembolization (TACE), transarterial radioembolization (TARE), stereotactic body radiotherapy (SBRT), and proton beam therapy (PBT) (4–27). Local treatments have the potential to improve survival by reducing the tumor burden and allowing subsequent systemic treatments to be more effective, but these treatments have a low level of evidence and could delay or interrupt systemic treatments. Thus, the ideal local treatment for patients with LMBC would be minimally invasive with a low morbidity and mortality rate and could minimize the delay or interruption of systemic treatments.

With technological advances in radiotherapy, SBRT has a growing role as non-invasive treatment option in the treatment for patients with metastatic liver tumors (26, 28, 29). In particular, the recent introduction of magnetic resonance imaging (MRI) guided radiotherapy has made it possible to precisely identify and verify the target before and/or during treatment (28). Although MRI guidance technology has not been applied to PBT yet, PBT has the potential to allow safe dose escalation in the target volumes while sparing uninvolved liver tissue due to the unique property of proton beams (called ‘Bragg peak’) when compared to radiotherapy with X-ray; moreover, PBT has been proven to be safe and effective as a local treatment for primary liver cancer (30–34). In addition, compared with conventional fractionated radiotherapy, hypofractionated radiotherapy has potential theoretical and practical advantages in terms of improvement of the therapeutic ratio by reducing cancer cell proliferation in involved tissues within the tolerance of surrounding uninvolved normal tissues and minimizing the delay or interruption of systemic treatments by shortening the overall duration of radiotherapy. Based on this background, hypofractionated PBT with various sequences and/or regimens of systemic treatments depending on the response to systemic treatments to LMBC and/or extrahepatic disease has been applied for patients with LMBC in our institution. The purpose of this study was to evaluate the clinical effectiveness of hypofractionated PBT for patients with LMBC.

## Material And Methods

### Patients

Patients with LMBC treated with proton beam therapy (PBT) between February 2013 and August 2019 were registered, and their database was reviewed. Treatment strategy was decided through discussion in a multidisciplinary team considering the patient’s performance status, location and size of tumor, and status of extrahepatic disease. Medical records (including admission information and summaries, discharge summaries, surgical notes, physician and nursing notes, laboratory reports, radiologic imaging and reports, and pathologic reports) of each patient were evaluated, and clinicopathologic data of each patient (such as age, histology, grade, clinical and pathological stage, pre-treatments prior to PBT, post-treatments after PBT, sites and times of disease progression, and follow-up data) were obtained. The collected data were managed by assigning case numbers anonymizing them, and then, data analyses were conducted according to the relevant guidelines and regulations. This study complied with the Declaration of Helsinki and Good Clinical Practice guidelines and was approved by the institutional review board of the National Cancer Center in Korea (20210266). Written informed consent was not required because the design of this study was retrospective.

### Treatment

**Figure 1:**
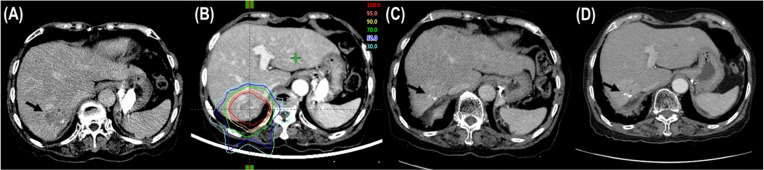
Tumor response after proton beam therapy (PBT). **(A)** CT scans prior to PBT showing the tumor (arrow). **(B)** The patient was treated with PBT. **(C)** CT scans at 6 months after PBT showing shrinkage of the tumor (arrow). **(D)** CT scans at 12 months after PBT showing complete response of the tumor (arrow).

The PBT procedures for liver tumors have been previously reported (30–34). In brief, a contrast-enhanced four-dimensional CT scan was obtained for each patient while monitoring the respiratory signals by a real-time position management system (Varian Medical Systems, Palo Alto, CA, USA). The gross tumor volume (GTV) was delineated in the average intensity projection CT images during exhalation (gated) phases (30% of the total respiratory cycle) fused with magnetic resonance imaging (MRI) and/or positron emission tomography (PET), and no margin was added for the clinical target volume (26, 27, 30). The internal target volume (ITV) and contours of organs at risk (OARs) were defined as the sum of the GTVs and each OAR in each CT image during gated (exhalation) phases, respectively, and the planning target volume (PTV) was defined as the ITV plus 5–7 mm margins in all directions. The PBT plan was designed with the intention of delivering 100% of the prescribed doses to at least 90% of the PTV using 2–3 (median, 3) non- or coplanar beams of 230 MeV passively double-scattered proton beams (Proteus 235; Ion Beam Applications, S.A., Louvain-la-Neuve, Belgium). Gray equivalent (GyE = proton physical dose [Gray] × relative biologic effectiveness [1.1]) was used to describe the radiation doses of PBT, and the median prescribed dose to the PTV was 66 GyE (range, 60–80) in 10 fractions, 5 times a week (**Figure 1**). Previously published dose-fractionations of PBT and dose-volume constraints for the OARs in liver and abdominal tumors were used (31, 32, 34–36). Briefly, dose-fractionations of PBT were decided by the tumor location as follow: i) 60GyE in 10 fractions was administered for tumor which was located within 2cm from gastrointestinal organs to avoid toxicity of gastrointestinal organs; ii) 66GyE in 10 fractions was administered for tumor which was located within 2cm from the hepatic hilum and more than 2cm from gastrointestinal organs; and iii) 70-80 GyE in 10 fractions was administered for tumor which was located more than 2cm from gastrointestinal organs and hepatic hilum. In dose-volume constraints for the OARs, the maximum dose for the spinal cord was <30 GyE; the radiation doses in 2 cm^3^ (D2cc) for the stomach, duodenum, and bowel were ≤39 GyE, ≤ 37 GyE, and ≤ 37 GyE, respectively; the relative liver volume receiving ≥27 GyE was limited to <60%; and the kidney volume receiving ≥18 GyE was <35%. Fasting for at least 4 hours prior to PBT was required for all patients at each treatment, and PBT radiation was delivered during gated phases after verifying each patient’s position and isocenter considering the liver, diaphragm, bones, etc., under the image guidance (AdapPT Insight; Ion Beam Applications, S.A., Louvain-la-Neuve, Belgium) using X-ray and/or cone beam CT images.

### Assessments and Statistical Analysis

Follow-up examinations with routine laboratory tests, tumor markers, and contrast-enhanced CT and/or MRI were performed at 1 month after PBT and every 3-4 months thereafter. Disease progression was determined by pathologic and/or radiological findings showing an increase in size over time. The appearance of regrowth or a new tumor within the PTV to target lesion(s) was defined as local progression, while the appearance of regrowth of previously untreated nontarget lesion(s) or a new tumor within the liver outside of the PTV and at extrahepatic sites was defined as intrahepatic and extrahepatic progression, respectively. The tumor responses of LMBC(s) treated with PBT and the adverse events (AEs) related to PBT were assessed according to the Response Evaluation Criteria in Solid Tumors (RECIST) version 1.1 (37) and the Common Terminology Criteria for Adverse Events version 4.03 (https://ctep.cancer.gov/protocolDevelopment/electronic_applications/docs/CTCAE_4.03.xlsx), respectively. The freedom from local progression (FFLP), progression-free survival (PFS) and overall survival (OS) were calculated from the commencement date of PBT until the date of local progression, the date of any disease progression or death, and the date of death from any cause or the last follow-up (i.e., censoring), respectively. The probability of FFLP, PFS, and OS was calculated using the Kaplan–Meier method. The log-rank test was used to compare survival differences, and a *p value <*0.05 was considered statistically significant. All statistical analyses were conducted using STATA software (version 14.0; StataCorp, College Station, TX).

## Results

A total of 17 LMBC patients receiving PBT between February 2013 and August 2019 were registered. Patient characteristics at the time of PBT are summarized in **Table 1**. The median size of LMBCs treated with PBT was 2.4 cm (range, 1.0–4.0), and the number of LMBCs treated with PBT was one and two in 16 patients and 1 patient, respectively. Three patients (17.6%) had *de novo* LMBC(s), and 14 patients (82.4%) had LMBC(s) at disease progression (**Table 1**). The median interval between the occurrence of LMBC(s) and initial diagnosis was 33.1 months (range, 0–184.8). All patients received systemic treatments and/or radiofrequency ablation for a median of 16.2 months (range, 2.2–84.2) prior to PBT to LMBC(s) (**Table 1**). At the time of PBT to LMBC(s), four patients (23.5%) had only LMBC(s) treated with PBT, but 13 patients (76.5%) had more than one intrahepatic and/or extrahepatic metastatic disease(s) other than LMBC(s) treated with PBT (**Table 1**). After PBT to LMBC(s), subsequent maintenance treatments were continued in 13 patients (76.5%) until disease progression (**Table 2**).

**Table 1:** Patient characteristics.

Pt.	Age	Primary tumor stage	Molecular subtypes	Initial Tx prior to DP	TI to DP/LM from iDx (months)	Pre-Tx to DP/LM	Sites of DP outside the Targeted LM	Site of the Targeted LM	No. of Targeted LMs	Size of the Targeted LM (cm)
1	51	pT1cN0M0	Luminal B	BCS + RT + TMX	33.1/33.1	AT + GP	EHD/IHD	S6	1	3.1
2	60	pT1N1M0	HER2	MRM + CMF	19.4/49.9	RFA + TH + FAC + XL+ GP + N + TP + RMLL/+ TH	EHD/IHD	S4/8	1	3.0
3	56	pT2N0M0	Luminal B	MRM + CMF + TMX	52.7/184.8	AC + Torem + AT + GN + X + RLLW + Let/+ TP + GP	EHD	S7/8	1	4.0
4	74	pT1N0M0	HER2	MRM + + CMF + TMX	24.5/24.5	RFA + TH +Ex + TH + AT + T-DM1 + XL + Eri	IHD	S7	1	3.8
5	51	cT3N2M0 ypT3N2M0	HER2	AC + BCS + TH + RT + H/TMX	47.1/47.1	TH + H + RT + XL + N	EHD/IHD	S2/3	1	3.7
6	56	pT3N1M0	Luminal A	BCS + AC + T + RT + TMX	47.0/47.0	Let + T	No	S3	1	3.4
7	56	pT2N0M0	HER2	BCS + CMF + RT + H	10.2/10.2	T	No	S4	1	1.2
8	40	cT3N3M0 ypT3N3M0	HER2	AT + MRM + AT	3.9/21.2	Excision + RT + H/+ T-DM1	No	S4	1	1.0
9	50	pT1N0M0	HER2	BCS + RT + TMX	37.4/46.7	TH + TMX/+ Let + T-DM1 + FAC	EHD/IHD	S7	1	1.0
10	74	pT2N1M0	Luminal B	MRM + FEC + TMX	1.1/160.6	Ana + Let + Ex-Ev + Fulv + X + T+ TMX + RT/+PemVin	EHD	S8	1	3.0
11	59	cT3N1M0 ypT3N2M0	HER2	Let + MRM + AC + TH + RT + Let/H	18.7/18.7	XL	IHD	S8	1	2.4
12	47	cT4N2M1 ypT2NxM1	Luminal A	AC + MRM + TMX + T	0/91.6	Let + Ev-Ex + X	EHD/IHD	S6	1	2.4
13	43	cT2N1M1 ypT1N0M1	HER2	DHP + BCS +PH/TMX + RT	0/0	DHP + PH/TMX	No	S7	1	1.0
14	35	cT2N3M0 ypTisN1M0	HER2	TCHP + BCS + H + TMX + RT	22.2/22.8	DPH + RT	EHD/IHD	S4	1	1.6
15	51	pT3N3M0	Luminal B	MRM + AC + TMX	37.3/53.7	RT + T + Gem/+ Let + X + N	EHD	S8	2	3.2
16	51	cT2N2M1 ypT2NxM1	Luminal A	FAC + BCS + Let	0/0	–	IHD	S3	1	1.3
17	58	cT2N2M1	HER2	T-DM1 + TH + H	0/0	–	EHD/IHD	S7	1	1.0

Pt., patient; Tx, treatment; Pre-Tx, previous treatment; TI, Time interval, DP, disease progression; LM, liver metastasis; iDx, initial diagnosis; Dz, disease; EHD, extrahepatic disease; IHD, intrahepatic disease outside of the targeted LM; BCS, breast-conserving surgery, RT, radiotherapy, TMX, tamoxifen; Torem, toremifene; MRM, modified radical mastectomy; CMF, cyclophosphamide, methotrexate and fluorouracil; AT, doxorubicin and docetaxel; GP, gemcitabine and cisplatin; RFA, radiofrequency ablation; TH, docetaxel (paclitaxel) and trastuzumab; FAC, fluorouracil, doxorubicin and cyclophosphamide; FEC, fluorouracil, epirubicin and cyclophosphamide; XL, capecitabine, and lapatinib; N, vinorelbine; TP, paclitaxel and cisplatin; H, trastuzumab; T-DM1, trastuzumab emtansine; PemVin, pemetrexed and vinorelbine; Let, letrozole; Ana, anastrozole; Fulv, fulvestrant; Ev-Ex, everolimus-exemestane; Eri, eribulin; RMLW, right middle lung wedge resection; RLLL, right lower lung lobectomy; GN, gemcitabine and vinorelbine; DHP, docetaxel, trastuzumab and pertuzumab; TCHP, docetaxel, carboplatin, trastuzumab and pertuzumab.

**Table 2:** Treatment details and outcomes of patients with liver metastasis from breast cancer receiving proton beam therapy.

Pt.	TD (GyE)	Subsequent Tx prior to DP	Site(s) of DP	Subsequent Tx after DP	Tumor	LP	DP	Survival
	/fractions				response		(months)	
1	66/10	–	IHD	ICE + RFA + Ana	PR	7.5	4.6	DWD 14.4
2	66/10	–	EHD/IHD	RT	PR	–	1.7	DWD 11.5
3	66/10	–	EHD/IHD	Eri + N + Fulv	CR	–	3.5	DWD 32.1
4	70/10	–	EHD/IHD	Poz + FAC + XL + T-DM1	CR	–	7.4	DWD 39.3
5	60/10	–	EHD/IHD	Poz + Eri + GPH + Ate/H	PR	–	1.7	DWD 24.5
6	70/10	Ex-Ev	IHD	X + Eri + GP	CR	–	25.2	AWD 56.1
7	70/10	–	–	–	CR	–	–	NED 44.2
8	70/10	H	EHD	XL + Gem/pertuzumab	CR	–	16.9	AWD 37.8
9	70/10	Ex	EHD/IHD	RT + XL + Eri/H + N + GP + Ner + Fulv/Palbo	CR	–	1.5	AWD 36.6
10	70/10	GP	IHD	Fulv/Palbo + T	CR	–	19.7	AWD 25.4
11	70/10	XL	IHD	H + N + TP + TPH + Eri/H	CR	–	7.9	AWD 35.3
12	70/10	Eri	EHD/IHD	Fulv + Abe	CR	–	7.7	AWD 34.2
13	80/10	PH	–	–	CR	–	–	NED 29.6
14	66/10	PH	EHD/IHD	H + AC + Let + Fulv/Abe + X	CR	–	4.4	AWD 27.2
15	70/10	Ex-Ev	EHD/IHD	Eri + Fulv/Abe	CR	–	9.3	AWD 27.3
16	70/10	Let	–	–	SD	–	–	AWD 24.5
17	70/10	H	–	–	CR	–	–	AWD 24.0

Pt, patient; TD, total radiation dose; Tx, treatment; DP, disease progression; LP, local progression, IHD, intrahepatic disease; ICE, ifosfamide, carboplatin and etoposide; Poz; poziotinib; palbo, palbociclib; Abe, abemaciclib; Ner, neratinib; CR, complete response; PR, partial response; SD, stable disease; NED, no evidence of disease; DWD, death with disease; AWD, alive with disease; the other terms are the same as in **Table 1**.

**Figure 2:**
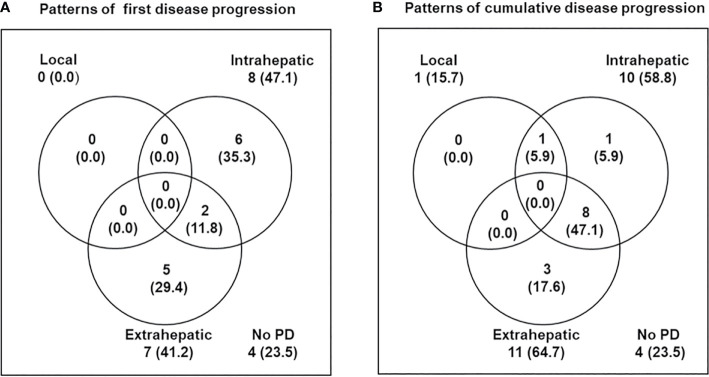
Patterns of disease progression. **(A)** First and **(B)** cumulative disease progression at the time of analysis.

**Figure 3:**
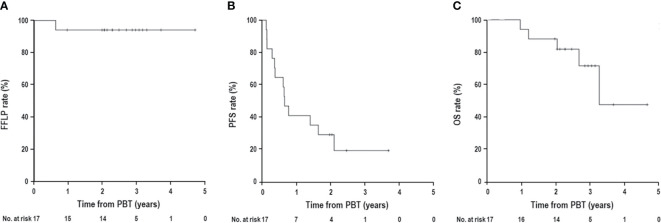
**(A)** Freedom from local progression (FFLP), **(B)** progression-free survival (PFS), and **(C)** overall survival (OS) curves in patients with liver metastasis from breast cancer receiving proton beam therapy (PBT).

The median follow-up time was 34.2 months (range, 11.5–56.1). During follow-up, the best tumor responses of LMBC(s) after PBT were complete response (CR) in 13 patients (76.5%), partial response (PR) in 3 patients (17.6%), stable disease (SD) in 1 patient (5.9%), and progressive disease (PD) in no patient (**Figure 1**). Of 17 patients, local progression of LMBC(s) treated with PBT was observed in 1 patient (5.9%) at 7.5 months after PBT, while local progression of LMBC(s) was not observed in the remaining 16 patients (94.1%) for a median follow-up time of 30.9 months (range, 11.5–56.1). The first sites of disease progressions were local in 0 patients (0%), intrahepatic in 8 patients (47.1%), and extrahepatic in 7 patients (41.2%), and the cumulative sites of disease progressions were local in 1 patient (5.9%), intrahepatic in 10 patients (58.8%), and extrahepatic in 11 patients (64.7%) (**Figure 2**). After the development of disease progression, subsequent salvage treatments were performed (**Table 2**). At the time of analysis, 12 patients were alive, and 5 patients died from disease progression. The median time of FFLP was not yet reached, and the 1-, 2-, 3-, and 4-year FFLP rates were 94.1% (95% confidence interval [CI], 82.9–105.3), 94.1% (95% CI, 82.9–105.3), 94.1% (95% CI, 82.9–105.3), and 94.1% (95% CI, 82.9–105.3), respectively (**Figure 3A**). The median time of PFS was 7.9 months (95% CI, 5.3–10.5), and the 1-, 2-, and 3-year PFS rates were 41.2% (95% CI, 17.8–64.5), 29.4% (95% CI, 7.6–51.1), and 19.6% (95% CI, -1.8–41.0), respectively (**Figure 3B**). The median OS time was 39.3 months (95% CI, 33.2–51.9), and the 1-, 2-, 3-, and 4-year OS rates were 94.1% (95% CI, 82.9–105.3), 88.2% (95% CI, 72.9–103.5), 71.7% (95% CI, 46.8–96.6), and 47.8% (95% CI, 6.1–89.5), respectively (**Figure 3C**). The median time of OS from the date of diagnosis of LMBC was 117.5 months, and the 1-, 2-, 3-, 4- and 5-year OS rates were 100%, 94.1% (95% CI, 82.9–105.3), 94.1% (95% CI, 82.9–105.3), 79% (95% CI, 57.6–100.4), and 79% (95% CI, 57.6–100.4), respectively. Patients who had only LMBC(s) treated with PBT (n=4) had significantly longer PFS than patients who had metastatic disease other than LMBC(s) treated with PBT (n=13) (2-year: 75.0% *vs*. 15.4%, *p*=0.034), while patients who had only LMBC(s) had a trend of higher OS than patients who had metastatic disease other than LMBC(s), but these findings were without statistical significance (2-year, 100% *vs*. 84.6%, *p*=0.074) due to the small size of the study population (n=17).

Within 3 months after PBT, 4 patients (23.5%) experienced dermatitis (grade 1, 3 [17.6%], and grade 2, 1 [5.9%]); two patients (11.8%) experienced grade 1 elevated alanine aminotransferase levels without evidence of disease progression; 3 patients (17.6%) experienced leukopenia (grade 1, 2 [11.8%], and grade 2, 1 [5.9%]); and one patient (5.9%) experienced grade 1 thrombocytopenia. Three months after PBT, 7 patients (41.2%) experienced grade 1 radiation pneumonitis, and one patient (5.9%) experienced grade 2 gastric ulcers managed by medication without invasive intervention. Treatment-related death and hepatic failure without evidence of disease progression were not observed in the subsequent follow-up period.

## Discussion

In breast cancer, tumor cells spread to the liver *via* the systemic circulation from the primary breast site; thus, isolated hepatic metastasis without multiorgan spread is rare, in contrast with colorectal cancer, in which tumor cells spread *via* the portal vein so that the liver is the first and principal metastatic site. Thus, systemic chemotherapy and/or hormonal treatments are currently the standard treatment for patients with LMBC, and even with recent advances in systemic treatments, the median OS time is 18-24 months. In addition, long-term survival of >5 years after systemic treatments alone has rarely been achieved (1). To date, the role of hepatic resection in patients with LMBC remains unclear, but this approach has been tried because hepatic resection is the only potentially curative treatment for primary or metastatic liver tumors. In a recent systemic review including 33 studies on hepatic resection of LMBC (n=965) (2), the median OS time was 35.1 months (range, 22.5–74), with a median 1-, 2-, 3-, and 5-year OS of 84.6% (range, 60–100), 71.4% (range, 40–90), 52.9% (range, 31-80), and 33% (range, 11.1-80), respectively. Although surgical series included a small, highly selected, and heterogeneous population, these results have suggested that despite LMBC being a systemic disease, local treatments have the potential to improve survival in selected patients who have LMBC confined to the liver or stable extrahepatic disease (3). However, hepatic resection is a potentially morbid procedure with a mortality rate of 0–5.9% and a median morbidity rate of 15% (2); thus, an effective local treatment option for LMBC that is less invasive and less influential on systemic treatments is needed.

Various nonsurgical local treatments, such as RFA, cryoablation, TACE, TARE, SBRT, and PBT, have been attempted (**Table 3**) (4–27). Local transarterial treatments, such as TACE (14–18) and TARE (14, 19–25), have been applied in LMBC patients with no or limited systemic treatment options or during holidays/breaks from systemic treatments. TACE has shown an objective response rate of 7-35.7% and a median OS time of 4.6-28.0 months (14–18); similarly, TARE has also shown an objective response rate of 28.9-56% and a median OS time of 4.0-13.6 months (14, 19–25) (**Table 3**). Although direct comparison among these studies is difficult due to different tumor burdens of intrahepatic and extrahepatic disease, patient characteristics, and selection criteria, these data suggest that transarterial local treatments, such as TACE or TARE, might be considered palliative treatment options rather than equivalent alternatives to hepatic resection for LMBC patients. Ablative treatments, such as RFA and cryoablation, are well-known minimally invasive and potentially curative local treatments for small sized (i.e., less than 2-3 cm) primary liver tumors, and they have also been applied for patients with LMBC (**Table 3**) (4–13). Ablative treatments of patients with LMBC have shown promising outcomes, with median OS times of 10.9–58.6 months and 1-, 3-, and 5-year OS rates of 68-90%, 25.3–49.3%, and 11–29%, respectively (**Table 3**). These results of ablative treatments suggest that ablative treatments are probably less effective at local tumor control than surgical resection, but they might be considered reasonable alternatives to surgical resection in selected patients who have LMBC confined to the liver or stable extrahepatic disease. However, the local progression rate after ablative treatment is relatively high, i.e., 7.3-53.8% (**Table 3**) (4–13). Even with complex planning, ultrasound and CT guidance and the use of multiple electrodes, the application and effectiveness of ablative procedures have been limited due to the size (i.e., >3 cm) of LMBC, proximity to major vessels and bile ducts, deep or subcapsular location, and visibility of the tumor with imaging guidance. Thus, another local treatment option for patients with LMBC is needed to overcome the technical limitations of ablative treatments and to achieve local tumor control comparable with that by hepatic resection.

**Table 3:** Studies on nonsurgical local treatments for liver metastasis from breast cancer.

			Tumor size, cm	EHD	CR/ORR	LP rate^*^	FFLP			OS				Adverse events
Authors	Modality	N	Median (range)	(%)	(%)	(%)	1-y (%)	3-y (%)	5-y (%)	Median	1-y (%)	3-y (%)	5-y (%)	(AEs)
Schullian et al. (10)	RFA	42	3.0 (1.0–9.0)	42.9	97.3/-	7.3	–	–	–	48.2	84.1	49.3	20.8	No major AEs
Bai et al. (4)	RFA	69	2.9^†^ (1.0-6.0)	46	92.6/-	11.6	–	–	–	26	81.8	25.3	11.0	1.1% major AEs
Kümler et al. (7)	RFA	32	2.0 (0.9–5.0)	47	-/-	22	–	–	–	33.5	87	48	–	3.1% G3 AEs
Tasci et al. (11)	RFA	24	3.4^†^ (1–10)	–	-/-	41.7	–	–	–	48	–	–	29.0	–
Carrafiello et al. (5)	RFA	13	3.5^†^ (0.5–7)	46.2	95/-	53.8				10.9	–	–	–	No major AEs
Jakobs et al. (6)	RFA	42	2.1^†^ (0.5-8.5)	41.9	96/-	13.5	–	–	–	58.6				4.6% major AEs
Meloni et al. (9)	RFA	52	2.5^†^ (0.7-5.0)	52	97/-	25.5	–	–	–	29.9	68	43	27	No major AEs
Veltri et al. (12)	RFA	45	2.3^†^ (1.0-4.5)	40	90/-	18	–	–	–	–	90	44	–	2.3% major AEs
Lawes et al. (8)	RFA	19	3.0^†^ (1.4-7.3)	57.9	63/-	15.8	–	–	–	–	–	–	–	–
Zhang et al. (13)	CRA	17	3.5 (2.0–5.0)	–	87.1/-	15.3	–	–	–	–	70.6	–	–	No major AEs
Vogl et al. (18)	TACE	208	-	23.4	0/13	–	–	–	–	18.5	69	40	33	No major AEs
Li et al. (16)	TACE	48	2.8^†^ (1-8)	39.6	7.1/35.7	–	–	–	–	28.0	63	13	–	No G3 AEs
Lin et al. (17)	TACE	23	16.5 (8.2-38.0)	69.6	0/26	–	–	–	–	16.9	–	–	–	34.8% G3 AEs
Eichler et al. (15)	TACE	43	–	49	0/7.0	–	–	–	–	13.6	–	–	–	7% G3 AEs
Chang et al. (14)	TACE	17	–	88.2	5.9/23.5	–	–	–	–	4.6	–	–	–	3% ≥G3 AEs
	TARE	30	–	66.7	0/40	–	–	–	–	12.9	–	–	–	0% ≥G3 AEs
Cianni et al. (19)	TARE	52	–	46.1	0/56	–	–	–	–	11.5	–	–	–	3.8% major AEs
Fendler et al. (20)	TARE	81	–	67	0/52	–	–	–	–	8.7	–	–	–	10% G3 AEs
Gordon et al. (21)	TARE	75	–	77	-/35.3	–	–	–	–	6.6	–	–	–	5.9% G3 AEs
Haug et al. (22)	TARE	58	–	66	-/-	–	–	–	–	4.0	–	–	–	3.8% mortality
Jakobs et al. (23)	TARE	30	–	57	0/61	–	–	–	–	11.7	–	–	–	3.3% mortality
Pieper et al. (24)	TARE	44	–	89	0/28.9	–	–	–	–	6.1	–	0	0	2.3% G3 AEs
Saxena et al. (25)	TARE	40	–	60	5/31	–	–	–	–	13.6	–	0	0	No G3 AEs
Onal et al. (26)	SBRT	22	1.6 (1.0–6.0)	68.2	58/90-	–	100	(88)^‡^	–	–	85	(57)^‡^	–	10% G3 AEs
Fukumitsu et al. (27)	PBT	8	4.0 (1.2–7.0)	0	-/-	–	86	86	86	–	88	73	58	No ≥G3 AEs
Present study	PBT	17	2.4 (1.0–4.0)	52.9	76.5/94.1	5.9	94.1	94.1	(94.1)^§^	39.3	94.1	70.8	(47.8)^§^	No ≥G3 AEs

N, number of patients; ORR, objective response rate (CR + PR); FFLP, free from local progression, OS, overall survival; y, year; RFA, radiofrequency ablation; CRA, cryoablation; TACE, transarterial chemoembolization; TARE, transarterial radioembolization; SBRT, stereotactic body radiotherapy; PBT, proton beam therapy; the other terms are the same as in **Tables 1** and **2**.

^*^Overall rate.

^†^mean.

^‡^2-year.

^§^4-year.

With technical advances in radiotherapy, a number of studies have shown that SBRT and PBT are effective and safe local treatment options for liver tumors (30–34, 38), but few studies have focused on SBRT and PBT for LMBC alone (26, 27). Onal et al. (26) analyzed 22 LMBC patients treated with both SBRT to LMBC and systemic treatment and reported promising outcomes in terms of 1- and 2-year FFLP rates of 100% and 88%, respectively, and 1- and 2-year OS rates of 87% and 57%, respectively. Fukumitsu et al. (27) analyzed 8 patients with LMBC without extrahepatic disease treated with PBT and reported 1-, 3- and 5-year FFLP rates of 86%, 86%, and 86%, respectively, and 1-, 3-, and 5-year OS rates of 88%, 73%, and 58%, respectively. In the present study, which analyzed 17 patients treated with PBT, the local progression rate was 5.9% with 1-, 3-, and 4-year FFLP rates of 94.1%, 94.1%, and 94.1%, respectively, and the median OS time was 39.3 months with 1-, 3-, and 4-year OS rates of 94.1%, 79.8%, and 47.8%, respectively. To date, there are no data from randomized study comparing PBT with SBRT, so it remains unanswered whether PBT is truly equivalent or superior to SBRT in these patients. In SBRT, the image guidance techniques using fiducials, surface guidance, stereotactic X-ray, CT, and MRI can allow a precise identification and verification of target before and/or during treatment and also reduce the target volume by minimizing the PTV margin (26, 28, 29, 38). MRI guidance technology is more helpful for tumors in the liver, frequently poorly visualized on the CT images, than those in other anatomical sites (28). To date, MRI guidance technology has not been applied to PBT in clinical practice, but several dosimetric studies comparing PBT with radiotherapy with X-rays in primary liver tumors showed that PBT can reduce the irradiated volume of remaining liver and allow dose escalation for tumors (39–41). Meta-analysis for primary liver tumors (42) showed a similar FFLP and OS and lower rate of toxicity in PBT compared to SBRT. In addition, although direct comparison of PBT with other local treatments was difficult due to heterogeneity of the study population among the studies, PBT yielded comparable or superior FFLP and OS to those of ablative treatments in previous studies (**Table 3**) (4–13) and comparable OS to that of hepatic resection (2).

The present study has several inherent limitations due to its relatively small (n=17) and retrospective nature. First, this study included a heterogeneous population with respect to factors such as tumor biology (i.e., status of hormonal receptors and human epidermal growth factor receptor), tumor burdens in intrahepatic and extrahepatic disease, responses to systemic treatments, and various histories of pre- and post-treatments; thus, potential selection bias and confounding factors related to prognosis were not thoroughly evaluated. In the present study, the patients who had only LMBC(s) treated with PBT had significantly longer PFS (2-year: 75.0% *vs*. 15.4%, *p*=0.034) and showed a trend toward longer OS (2-year, 100% *vs*. 84.6%, *p*=0.074) than patients who had other metastatic diseases. This finding implied that patients with LMBC confined to the liver or stable extrahepatic disease after systemic treatments may be subgroups that could benefit from local treatments, including PBT. Second, PBT for LMBC showed a safety profile, without ≥grade 3 AEs in the present study and other studies (27), but the interpretation of data should be carefully performed due to the probability of underestimation of AEs in retrospective studies due to the incompleteness of medical records, recall bias, etc. Third, the effect of the PBT dose on local tumor control was not evaluated due to the small size of the study population (n=17), but in the present study, PBT showed promising outcomes in terms of a local progression rate of 5.9% and 3-year FFLP rates of 94.1%. In addition, to the best of our knowledge, few reports of PBT for LMBC with a smaller study population (n=8) than that of the present study are available to date (27). However, further, large-scale studies are needed to confirm the effectiveness of PBT for LMBC and to select patients who will benefit from PBT.

In conclusion, the present study showed that PBT can yield promising FFLP and OS rates similar to those resulting from hepatic resection and ablative treatments in patients with LMBC, with a safe profile of toxicity. These findings suggest that PBT may be considered a local treatment option for patients with LMBC confined to the liver or stable extrahepatic disease after systemic treatments.

## Data Availability Statement

All data used in this study are available upon request to the corresponding author.

## Ethics Statement

This study was approved by the Ethical Committee of the National Cancer Center (20210266), and written informed consent for participation was not required for this study in accordance with the national legislation and the institutional requirements.

## Author Contributions

TK, KL, and SS: conceptualization. TK, KL, and SS: data collection. TK: formal analysis. TK, KL SS, Y-JK, DK, HC, E-GL, JH, SJ, SL, HK and ESL: investigation. TK: writing the original draft. TK, KL, and SS: supervision. All authors contributed to the article and approved the submitted version.

## Funding

This study was supported by National Cancer Center Grants (2110351 and 1910300). The funders had no role in the design of this study; curation, analysis, and interpretation of data; writing of the manuscript; or decision to publish this study.

## Conflict of Interest

The authors declare that the research was conducted in the absence of any commercial or financial relationships that could be construed as a potential conflict of interest.

## Publisher’s Note

All claims expressed in this article are solely those of the authors and do not necessarily represent those of their affiliated organizations, or those of the publisher, the editors and the reviewers. Any product that may be evaluated in this article, or claim that may be made by its manufacturer, is not guaranteed or endorsed by the publisher.
